# Met Receptor Tyrosine Kinase Signaling Induces Secretion of the Angiogenic Chemokine Interleukin-8/CXCL8 in Pancreatic Cancer

**DOI:** 10.1371/journal.pone.0040420

**Published:** 2012-07-17

**Authors:** Kristen S. Hill, Ivana Gaziova, Lindsay Harrigal, Yvette A. Guerra, Suimin Qiu, Sarita K. Sastry, Thiruvengadam Arumugam, Craig D. Logsdon, Lisa A. Elferink

**Affiliations:** 1 Department of Neuroscience and Cell Biology, University of Texas Medical Branch, Galveston, Texas, United States of America; 2 Department of Pathology, University of Texas Medical Branch, Galveston, Texas, United States of America; 3 Department of Biochemistry and Molecular Biology, University of Texas Medical Branch, Galveston, Texas, United States of America; 4 UTMB Cancer Center, University of Texas Medical Branch, Galveston, Texas, United States of America; 5 Department of Cancer Biology, University of Texas M. D. Anderson Cancer Center, Houston, Texas, United States of America; University of Nebraska Medical Center, United States of America

## Abstract

At diagnosis, the majority of pancreatic cancer patients present with advanced disease when curative resection is no longer feasible and current therapeutic treatments are largely ineffective. An improved understanding of molecular targets for effective intervention of pancreatic cancer is thus urgent. The Met receptor tyrosine kinase is one candidate implicated in pancreatic cancer. Notably, Met is over expressed in up to 80% of invasive pancreatic cancers but not in normal ductal cells correlating with poor overall patient survival and increased recurrence rates following surgical resection. However the functional role of Met signaling in pancreatic cancer remains poorly understood. Here we used RNA interference to directly examine the pathobiological importance of increased Met signaling for pancreatic cancer. We show that Met knockdown in pancreatic tumor cells results in decreased cell survival, cell invasion, and migration on collagen I *in vitro*. Using an orthotopic model for pancreatic cancer, we provide *in vivo* evidence that Met knockdown reduced tumor burden correlating with decreased cell survival and tumor angiogenesis, with minimal effect on cell growth. Notably, we report that Met signaling regulates the secretion of the pro-angiogenic chemokine interleukin-8/CXCL8. Our data showing that the interleukin-8 receptors CXCR1 and CXCR2 are not expressed on pancreatic tumor cells, suggests a paracrine mechanism by which Met signaling regulates interleukin-8 secretion to remodel the tumor microenvironment, a novel finding that could have important clinical implications for improving the effectiveness of treatments for pancreatic cancer.

## Introduction

Pancreatic ductal adenocarcinoma (PDAC) is an aggressive cancer with a median patient survival rate of less than one year, making it the fourth leading cause of cancer deaths in the United States [1]. The high mortality rate of PDAC patients is due to several factors. In the absence of effective screening methods, 80–85% of patients present with advanced disease that often precludes curative resection [2]. Furthermore, standard treatments for advanced disease are largely ineffective [3], [4]. Thus there is an urgent need to understand the molecular basis of PDAC growth to identify targets of high therapeutic value. Recent genetic analysis of pancreatic tumors identified several genetic mutations common to 75–90% of patient cases important for PDAC initiation and subsequent development of preinvasive pancreatic intraepithelial neoplasms (PanINs 1–3) [5]–[7]. In agreement with this, genetically engineered mouse models for PDAC have confirmed the role of specific genetic mutations in the initiation and development of early stage disease. Examples are PanIN-1 (Kras activation, loss of Notch2) [8], [9], PanIN-2 (loss of function mutations in tumor suppressor p53 and p16INK4a) [10] and PanIN-3 (inactivation of p53, SMAD4/DPC4 and BRCA2) [8], [11], [12]. In contrast to these early stage preinvasive lesions, PDAC has a lack of defined mechanisms.

Several signaling pathways are likely involved in PDAC. One candidate is the Met/Hepatocyte Growth Factor (HGF) signaling axis. Under physiological conditions, the Met receptor tyrosine kinase and its ligand HGF are expressed at low levels in pancreatic acinar cells and the stromal compartment respectively [13]–[15]. Paracrine binding of HGF to Met results in receptor phosphorylation leading to increased cell survival and motility [16], [17]. In contrast to lung and gastric adenocarcinomas in which activating mutations prolong Met signaling, such gain-of–function Met mutations have yet to be identified in PDAC. Normal pancreatic ducts express low Met levels. Conversely Met is over-expressed in up to 80% of PDAC cases [18], is a strong indicator for increased recurrence rates and overall poor PDAC patient survival [13], [19]–[22]. Met expression is present in up to 29 tumorigenic pancreatic cell lines with different genetic backgrounds, including ASPC-1, Panc-1, BxPC-3 and Suit-2 cells [13], [23]. One exception is the poorly differentiated MIA PaCa-2 cell line, which does not express endogenous Met [13], [23]. Recently, the Met small molecule inhibitor SGX523 was reported to reduce the growth and infiltration of subcutaneous pancreatic tumors [24] raising interest in Met as a potential therapeutic target for advanced disease. However, the precise role of Met signaling for PDAC remains unresolved.

In this study, we used RNA interference to reduce Met signaling in human pancreatic xenografts using an *in vivo* mouse orthotopic model. Met knockdown (MetKD) cells remained competent for forming orthotopic pancreatic tumors *in vivo*; however, the resulting MetKD xenografts were significantly growth inhibited relative to tumors resulting from control cells expressing a non targeting (NT) shRNA. Immunohistochemical analysis of MetKD xenografts showed increased cell apoptosis accompanied by reduced cell proliferation and mean vessel density (MVD in the periphery of MetKD xenografts. Consistent with this scenario, we show that Met knockdown reduces secretion of interleukin-8/CXCL8 (IL-8), a potent pro-angiogenic chemokine that functions as a paracrine regulator of endothelial cell proliferation, an activator of neutrophils and a chemoattractant for fibroblasts and other immune cells [25]. Our finding that Met is an upstream regulator of IL-8 secretion is significant and suggests one mechanism by which Met signaling could regulate pancreatic cancer *in vivo*, a novel finding that may provide unique opportunities for clinical intervention.

## Materials and Methods

### Ethics Statement

All animals were cared for in accordance with the Office for Protection from Research Risks (OPRR) and Animal Welfare Act guidelines under an animal protocol approved by the University of Texas M. D. Anderson Institutional Animal Care and Use Committee. This study was approved by the University of Texas M. D. Anderson Institutional Animal Care and Use Committee.

### Antibodies, Cell Lines, and Maintenance

An antibody against the C-terminus of Met (C-28) was purchased from Santa Cruz Biotechnology. A mouse monoclonal antibody against β-actin was purchased from Sigma. Site-specific anti-phospho tyrosine antibodies for Met Y1234/1235 were from Upstate. An antibody specific for the extracellular domain of Met (anti-hHGFR) was purchased from R&D Systems, Inc. Antibodies against Ki67 were purchased from Abcam, cleaved caspase-3, ERK1/2, and phospho ERK1/2 T202/Y204 (pERK) antibodies, AKT and phospho AKT (S473) were from Cell Signaling. CD31 antibodies were purchased from PharMingen and recombinant human and murine HGF from PeproTech. ASPC-1, BxPC-3 and MIA PaCa-2 cells were purchased from ATCC and DNA fingerprints for each line verified by site-specific PCR (Seqwright). BxPC-3 cells were maintained in RPMI medium 1640 (Gibco) with 10% Fetal Bovine Serum (FBS) and 1× Penicillin/Streptomycin. ASPC-1 cells were maintained in Dulbecco’s modified Eagles medium (DMEM: Gibco) with 10% FBS and 1× Penicillin/Streptomycin. MIA PaCa-2 cells stably expressing exogenous wild type Met have been described elsewhere [23]. 293FT cells (Invitrogen) were co-transfected with lentiviral envelope (pMD2G), packaging (psPAX2), and shRNA plasmids directed against human Met (Sigma) to produce lentiviruses encoding the shRNA constructs. Using flow cytometry we screened four lentiviral plasmids that encoded unique shRNA sequences directed against the coding region or the 3′UTR of human Met and examined their ability to knockdown Met expression. We identified two shRNA viruses (#2, Sigma NM_000245.2-3702s1c1 and #5, Sigma NM_000245.2-4462s1c1) that routinely resulted in reduced Met expression (not shown). BxPC-3 and ASPC-1 cells plated onto tissue culture dishes were infected with serial dilutions of lentivirus encoding MetKD shRNA-2 (5′-CCGGAGACTCATAATCCAACTGTAACTCGAGTTACAGTTGGATTATGAGT CTTTTTTG-3′), MetKD shRNA-5 (5′-CCGGGCACTATTATAGGACTTGTATCTCGAGATACAAGTCTTATAATAGTGCTTTG-3′) or NT control shRNA (5′-CCTAAGGTTAAGTCGCCCTCGCTCGAGCGAGGGCGACTTAACCTTAGG-3′), in the presence of 8 µg/mL hexadimethrine bromide prior to selection with 5 µg/mL puromycin for 7 days, and colonies were maintained in media containing 2.5 µg/mL puromycin for 2 months. pCDNA plasmids encoding CXCR1 and CXCR2 were a kind gift from Xavier Navarro, UTMB Galveston.

### Flow Cytometry

1–2×10^6^ cells were seeded onto 10 cm tissue culture plates and following adhesion were serum-starved overnight. Flow cytometry for surface stained Met was performed using a BD FACS Array as previously described [23].

### Anchorage Independent Growth Assays

A bottom layer of 1% agarose diluted in media containing 1% FBS with or without 100 ng/mL HGF (PeproTech) was deposited onto 4 well tissue culture plates. After the bottom layer solidified at room temperature for 30 min, 5×10^3^ cells were resuspended in media containing 1% FBS with or without 100 ng/mL HGF and a final concentration of 0.4% agarose. The cell and agarose mixture was overlaid onto the tissue culture plates containing 1% agarose and allowed to solidify at 4°C for 10 min. Once solidified, media containing 1% FBS with or without HGF was added and cells grown for 6 weeks in a 37°C incubator with 5% CO_2_. Media supplemented with HGF was changed every 2–3 days.

### Cell Invasion and Migration Assays

Cell migration and invasion assays were performed as previously described [26]. Wound healing assays were performed as previously described [23]. Images were captured using an ECLIPSE TE2000-U inverted microscope (Nikon) and analyzed using MetaMorph v7.3.1. (Molecular Devices). For live cell migration on collagen I, cells were serum-depleted overnight (1% FBS) and then plated onto 35 mm glass bottom tissue culture plates pre-coated with 15 µg/mL rat tail collagen I (BD Bioscience) overnight at 4°C. Cells were allowed to adhere for 1 hr before treatment with media containing 1% FBS alone or with 100 ng/mL HGF. Plates were placed in a BioStation Live Cell Imaging System (Nikon) and the chamber temperature was allowed to equilibrate for 20 min, after which images of each field were collected every 2 min for at least 2 hr. Cell migration was assessed as the total path length in micrometers traveled and cell velocity (microns/min) was determined using NIS Elements Software (AR3.10).

### Mice and Orthotopic Tumor Studies

ASPC-1 (5×10^5^) or BxPC-3 (1×10^6^) NT, MetKD-2 or MetKD-5 cell lines expressing equivalent levels of firefly luciferase, were injected in 50 µL of PBS into the body of the pancreas of 5–6 week old male athymic nude mice (NCI-Charles River) as previously described [26]. Bioluminescence imaging was conducted every 10 days using a cryogenically cooled IVIS 100 imaging system coupled to a data acquisition computer running Living Image Software (Xenogen Corp) as previously described [26]. Each animal was normalized to its signal intensity at day 10 to adjust for variations in initial tumor seeding and is reported as the fold-increase in tumor volume measured as the number of photons emitted from the tumor per sec per cm^2^. At necropsy, primary tumors were surgically removed and weighed, and tissues either fixed with formalin for histological evaluation or flash frozen for further analysis.

### Quantitative Reverse Transcription PCR

Flash frozen xenografts were homogenized in trizol reagent (Invitrogen) and total RNA isolated according to manufactures directions. 500 ng of RNA was converted to cDNA using iScript cDNA synthesis (BioRad) with random hexamer primers. 2 µL of the cDNA was used as the template for relative quantitative real time PCR using SYBR Green PCR Master Mix (Applied Biosystems). SYBR green incorporation was measured over 40 cycles of 95°C for 30 sec, 60°C for 30 sec and 72°C for 1 min using primer sequences for Met (forward 5′ TAAGTGCCCGAAGTGTAAGC -3′ and reverse 5′- CTTGCCATCATTGTCCAACAAAGTCCC -3′) and GADPH (forward 5′-CAATGACCCCTTCATTGACCTC-3′ and reverse 5′-AGCATCGCCCCACTTGATT-3′). The level of Met mRNA was determined by normalizing the C_T_ value to the C_T_ of human GAPDH using the comparative C_T_ (ΔΔ*C*_T_) method as described by the manufacturers (Applied Biosystems). For studies using cell lines, cDNA was synthesized from total RNA using Accuscript (Stratagene) and PCR performed using AmpliTaq Gold PCR master mix (applied Biosystems) using the following primer sets for human CXCR1, 5′-TGGGAAATGACACAGCAAAA-3′ (forward) and 5′-AGTGTACGCAGGGTGAATCC-3′ (reverse), human CXCR2, 5′-ACTTTTCCGAAGGACCGTCT-3′ (forward) and 5′-GTAACAGCATCCGCCAGTTT-3′ (reverse), human GADPH, 5′-ACGCATTTGGTCGTATTGGG-3′ (forward) and 5′-TGATTTTGGAGGGATCTCGC-3′ (reverse) were used. Amplified products were resolved on 2% agarose gels containing ethidium bromide and imaged using an AlphaInnotech gel documentation system (AlphaInnotech).

#### Immunohistochemistry

Orthotopic tumors were processed for immunohistochemistry as previously described [26], [27] using antibodies against Met (C-28), Ki67 or cleaved caspase-3. All slides were counterstained with hematoxylin, and blinded so that scoring was performed in an unbiased manner. Proliferation and apoptosis were quantified by determining the percentage of cells that were positive for Ki67 and cleaved caspase-3 respectively. Three random fields per tumor section where imaged using a 40× objective on a Zeiss Axiovert 200 microscope with a Zeiss MRc5 color camera. The number of positively stained (C_P_) and negative unstained (C_N_) cells were counted using MetaMorph v7.3.1 (Molecular Devices). The percentage of positive staining (%P) cells was determined using the following equation: %P =  (C_P_/(C_P_+C_N_))*100. The percentage of necrosis (%N) was determined in H&E stained xenograft tumor specimens imaged using a 5× objective and analyzed using MetaMorph v7.3.1. The percentage of necrosis (%N) for each tumor specimen was calculated using the following equation %N = (A_N_/A_T_)*100, where A_N_ and A_T_ represent the necrotic and total areas respectively. CD31/platelet endothelial cell adhesion molecule 1 (PECAM-1) staining was assessed in frozen pancreatic tissues that were sectioned (8–10 µm), mounted on positively charged slides and air-dried for 30 min and stained using CD31 antibodies as described previously [28]. Control samples exposed to a secondary antibody alone showed no specific staining. For the quantification of mean vessel density (MVD) in sections stained for CD31, 3 fields per tumor section were imaged using a 20× objective on a Zeiss Axiovert 200 microscope and the number of discrete CD31 positive vessels per field were scored.

### Angiogenesis Arrays and ELISA

3×10^6^ cells were seeded onto a 10 cm tissue culture plate and cells were allowed to adhere overnight in a 37°C humidified incubator with 5% CO_2_. Cells were washed in media with 1% FBS and serum-depleted overnight prior to treatment with DMEM containing 1% FBS alone (no ligand) or with 100 ng/mL HGF for 48 hr. Conditioned media was collected and centrifuged to remove any cell debris prior to analysis. Human Angiogenesis Arrays, VEGF (R&D Systems, Inc) and human IL-8 ELISAs (BD Biosciences) were performed on conditioned media as outlined by the manufacturer. To quantify tumor VEGF and IL-8 levels, snap frozen tumor samples were processed as previously described [29]. ELISA was performed using 10 µg protein diluted in 100 µL with RIPA buffer (150 mM NaCl, 50 mM Tris-HCl pH 7.4, 0.1% SDS, 1% NP-40 (Igepal), 0.5% deoxycholic acid) containing protease (2 mg/mL Aprotinin, 2 mg/mL Pepstatin A, and 2 mg/mL Leupeptin) and phosphatase inhibitors (10 mM NaF, 2 mM Na_3_VO_4_). VEGF and IL-8 protein levels detected in xenografts by ELISA are reported as pg IL-8 or VEGF per µg protein lysate (BCA Assay, Pierce).

### Statistical Analysis

All statistical analyses were performed using GraphPad Prism software (v4), using a One or Two-way analysis of variance (ANOVA) with a Bonferroni post-hoc analysis to determine statistical significance between groups.

## Results

### Stable Knockdown of Met

**Figure 1:**
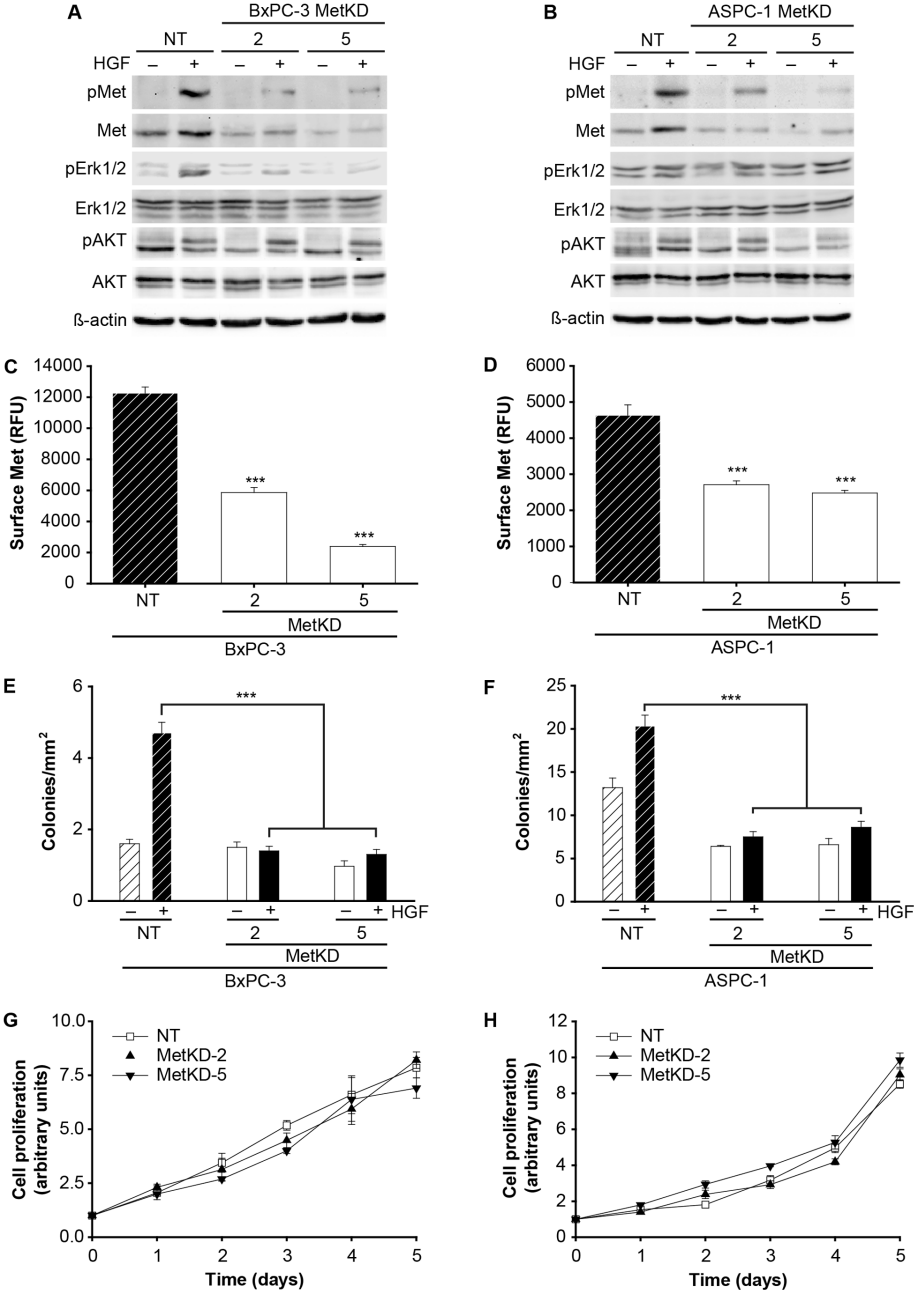
Met knockdown reduces BxPC-3 and ASPC-1 cell survival. BxPC-3 (A) or ASPC-1 (B) cells infected with recombinant lentivirus expressing Met knockdown shRNAs (2 or 5) or a non targeting (NT) shRNA were treated without (-) or with (+) HGF and examined by Western analysis for pMet (Y1234/1235), Met, pErk1/2, Erk1/2, pAKT, AKT and β-actin levels (n = 3). Flow cytometry detected reduced Met surface expression in BxPC-3 (C) and ASPC-1 (D) MetKD cells relative to NT cells. Values are expressed as the average mode +/− SEM (***p<0.001; ANOVA, n = 3). Reduced anchorage independent growth of BxPC-3 (E) and ASPC-1 (F) MetKD cells in soft agar relative to NT cells (***p<0.001; ANOVA, n = 3). Met knockdown had minimal effect on the growth of BxPC-3 (G) and ASPC-1 (H) MetKD cells relative to NT cells.

Pharmacological inhibitors support an oncogenic role for increased Met signaling in several solid tumors. However, direct evidence supporting the pathological significance of increased Met levels for PDAC remains unresolved. Accordingly, we used RNA interference to knockdown Met in the human pancreatic cell lines BxPC-3 and ASPC-1, as they are derived from primary human pancreatic cancers and express high levels of wild type Met. Whereas BxPC-3 cells are wild type for Kras and express mutant p53, ASPC-1 cells are mutant for Kras and p53, key mutations important for cellular transformation and the initiation of early stage disease [30]. Two polyclonal populations of Met knockdown (MetKD) cells expressing different Met specific shRNA sequences (MetKD-2 and MetKD-5) were established using recombinant lentiviruses. Polyclonal populations of ASPC-1 or BxPC-3 cells expressing a NT shRNA were used as controls. Western analysis confirmed stable knockdown of total Met and HGF activated Met in BxPC-3 and ASPC-1 MetKD-2 and MetKD-5 cells relative to NT cells. pERK was detected only in HGF-treated BxPC-3 cells, consistent with the wild type Kras status of these cells. As expected, lower levels of HGF-stimulated pERK were detected in BxPC-3 MetKD cells when compared to NT expressing cells (Figure 1A). Conversely, western analysis detected high levels of pERK in HGF-treated and untreated ASPC-1 Met KD and NT cells consistent with the oncogenic Kras status of these cell lines (Figure 1B). Comparable pAKT levels were routinely detected in response to HGF in MetKD and NT cells. Flow cytometry analysis confirmed reduced expression of cell surface Met in the MetKD BxPC-3 and ASPC-1 cells when compared to control NT cells (Figure 1C & 1D).

To assess whether Met signaling affects the tumorigenic potential of BxPC-3 and ASPC-1 cells, we performed anchorage independent growth assays in soft agar. NT and MetKD cells were seeded into 0.4% agarose in the presence or absence of HGF and the number of resulting colonies, as defined as a cluster of three or more cells was scored. In NT cell lines, HGF treatment resulted in a significant increase in the number of colonies present in soft agar consistent with a role for Met signaling in anchorage independent cell growth. Conversely, HGF-treated MetKD cells showed reduced capacity to grow in an anchorage independent manner in soft agar (Figure 1E & 1F). We next examined the effect of Met knockdown on HGF-induced cell proliferation. Interestingly, HGF-induced proliferation was not affected by Met knockdown in BxPC-3 and ASPC-1 cells (Figure 1G & 1H).

**Figure 2:**
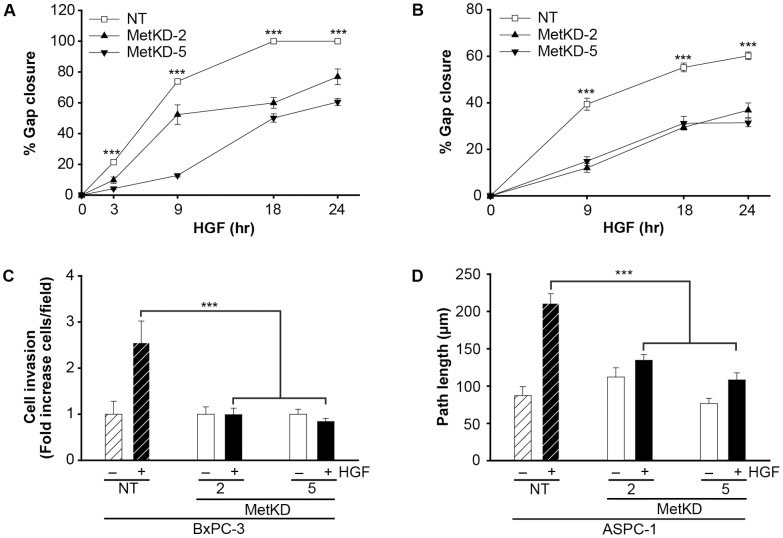
Decreased cell motility and invasion *in vitro* by Met knockdown. Serum-depleted, confluent NT or MetKD BxPC-3 (A) or ASPC-1 (B) cells were wounded with a scratch, six regions marked, and immediately imaged (0 hr) prior to treatment with HGF. Identical regions along the scratch were imaged after 3, 9, 18, and 24 hr and the values were normalized to the initial size of the scratch at 0 hr. Cell migration is reported as the % gap closure at each time point (***p<0.001; ANOVA, n = 3). MetKD reduces BxPC-3 cell invasion in response to HGF relative to control cells (C). Values represent the fold change in the number of cells/field and are reported as the mean +/− SEM (***p<0.001; ANOVA, n = 3). Live cell imaging of ASPC-1 MetKD cells plated on collagen I-coated glass bottom cell culture dishes show decreased cell migration in response to 100 ng/mL HGF (D). Images were collected every 2 min for a total of 120 min and data is expressed as the mean path length +/− SEM per condition (***p<0.001; ANOVA, n>30 cells).

The effect of Met knockdown on HGF-induced cell migration was examined using a wound healing assay. As shown in Figs 2A & B (Supporting Information S1), MetKD cells consistently showed reduced HGF-induced motility compared to NT cells. Using a matrigel-based invasion assay, we also show that loss of Met signaling significantly reduced the invasive phenotype of BxPC-3 MetKD cells (Figure 2C). PDAC is characterized by an abundant desmoplastic stroma that is highly enriched in extracellular matrix components including collagen I. As a result, we performed live cell imaging studies to measure differences in HGF-induced migration of ASPC-1 NT and MetKD cell lines seeded on collagen I (Figure 2D). Cells were allowed to adhere for 1 hr on collagen I prior to treatment with HGF, after which live cell migration was examined by measuring the total path length of migrating cells over a 2 hr period. In the absence of HGF, comparable migratory path lengths were detected for ASPC-1 MetKD and NT cells (NT: 87.2 µm +/−12.0; MetKD-2: 112.1 µm +/−12.3; MetKD-5: 76.5 µm +/−7.0) (Figure 2D). Treatment with HGF increased the path length of APSC-1 NT cells (216.7 µm +/−13.1), correlating with increased cell velocity (data not shown). Conversely, HGF treatment of MetKD-2 and MetKD-5 cells did not result in increased path length (Movie S1, Movie S2, Movie S3). Consequently, Met knockdown results in decreased ASPC-1 cell migration on collagen I.

### Met Knockdown Impairs *in vivo* Tumor Growth

**Figure 3:**
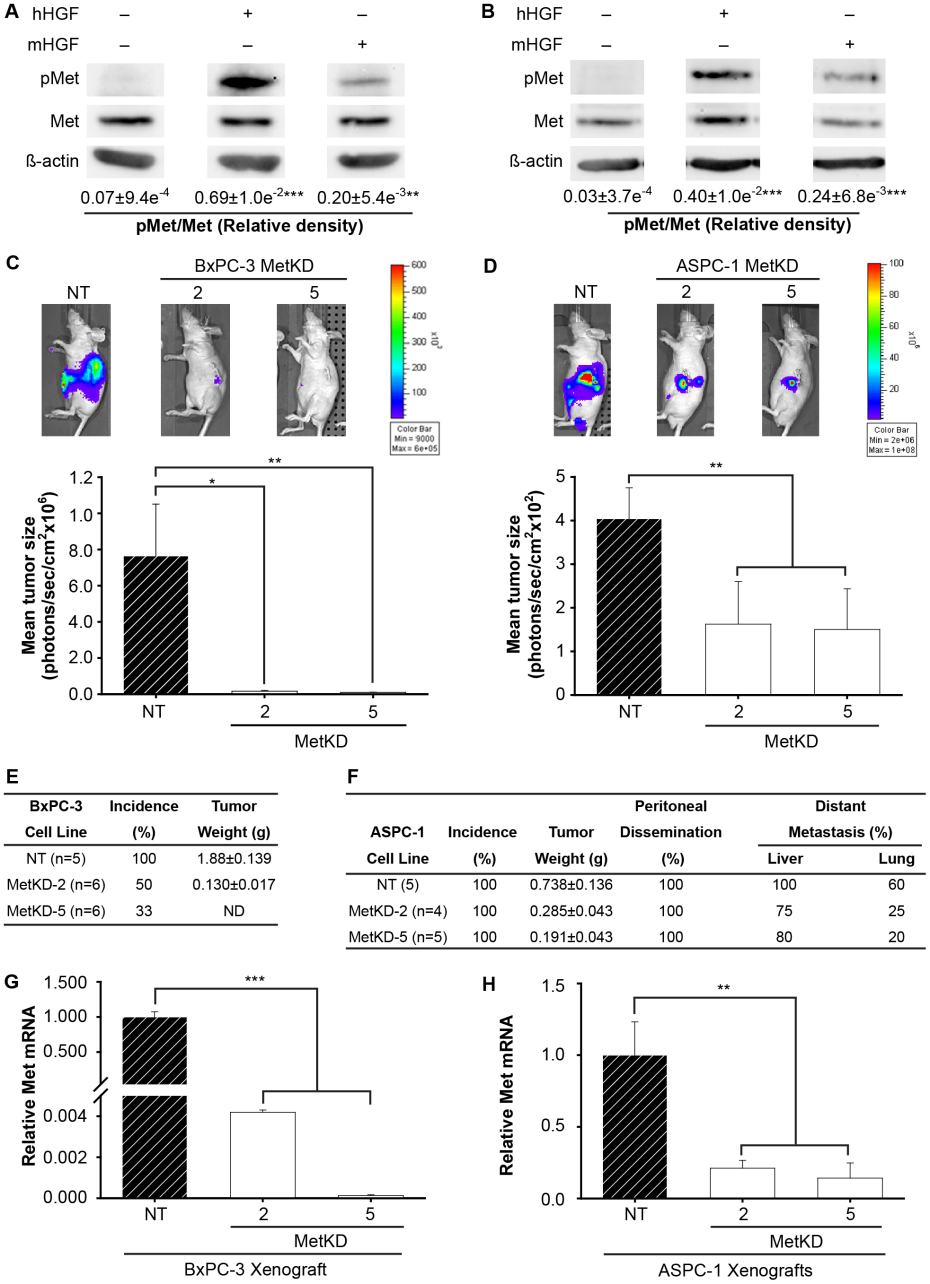
Met impairs *in vivo* tumor burden. Western analysis confirmed that murine (mHGF) and human HGF (hHGF) activate human Met in BxPC-3 (A) and ASPC-1 (B) NT cell lines. No Met activation was observed in the absence of ligand. Values are expressed as mean relative density of pMet/Met +/− SEM (**p<0.01 ***p<0.001; ANOVA, two separate experiments). Tumor size was measured every 10 days using *in vivo* bioluminescent imaging for BxPC-3 (C) or ASPC-1 (D) cells post injection and are reported as mean tumor size in photons/sec/cm^2^ (*p<0.05, **p<0.01; ANOVA). Representative bioluminescent images are shown immediately prior to necropsy at 30 days (BxPC-3) or 45 days (ASPC-1). The weight and overall incidence (%) of BxPC-3 primary tumors (E) and ASPC-1 (F) primary and metastatic tumors are listed. Stable knockdown of Met mRNA in BxPC-3 (G) and ASPC-1 (H) MetKD xenografts was confirmed using qRT-PCR. Data were normalized to GADPH and are reported relative to the respective NT control (**<p0.01; ***p<0.001; ANOVA, n = 2).

We sought to examine the consequence of Met disruption on tumor growth *in vivo*, using an orthotopic mouse model for pancreatic cancer. Since cross species differences in the interaction of murine HGF with human Met has been reported [31], we first used Western analysis to confirm the activation of human Met by murine HGF in BxPC-3 and ASPC-1 cells. Using conditions that result in maximal Met phosphorylation by human HGF, we show that human Met was activated by the murine and human ligand using site specific anti-Met phospho-tyrosine antibodies and Western analysis (Figure 3A & 3B). Under these conditions human HGF was 3–5 fold more effective than murine HGF in inducing Met phosphorylation, consistent with previous reports that murine HGF is functionally active towards the human receptor *albeit* with a lower affinity [32], [33].

To examine the effect of Met knockdown on PDAC growth, we used BxPC-3 and ASPC-1 cells pre-labeled with firefly luciferase to follow tumor burden non-invasively [26]. Equal numbers of MetKD and NT control cells were injected into the body of the pancreas of athymic nude mice and tumor burden quantified using *in vivo* bioluminescence [26]. Strong tumor growth was detected in mice injected with control cells expressing the NT shRNA (Figure 3C & 3D). Conversely, tumor incidence and burden was significantly reduced in mice injected with MetKD BxPC-3 or ASPC-1 cells. At necropsy (40 and 45 days for BxPC-3 and ASPC-1 injected mice respectively), small tumors masses were readily detected in pancreata injected with BxPC-3 MetKD-2 cells, but not with BxPC-3 MetKD-5 cells (Figure 3C and 3E). This was not due to problems with tumor seeding, as small tumor fields were readily detected microscopically in H&E stained sections of MetKD tumors consistent with the bioluminescence data (Supporting Information S2). In mice injected with ASPC-1 cells, MetKD cells show reduced primary tumor size in the orthotopic model compared to mice injected with NT cells (Figure 3D), correlating with decreased incidence of local (liver) and distant (lung) tumor metastasis (Figure 3F). qRT-PCR confirmed reduced Met mRNA levels in MetKD tumors when compared to NT tumors (Figure 3G & 3H). Thus, loss of Met signaling in BxPC-3 and ASPC-1 cells impairs tumor burden *in vivo*.

### Met Signaling Remodels the Tumor Vasculature

**Figure 4:**
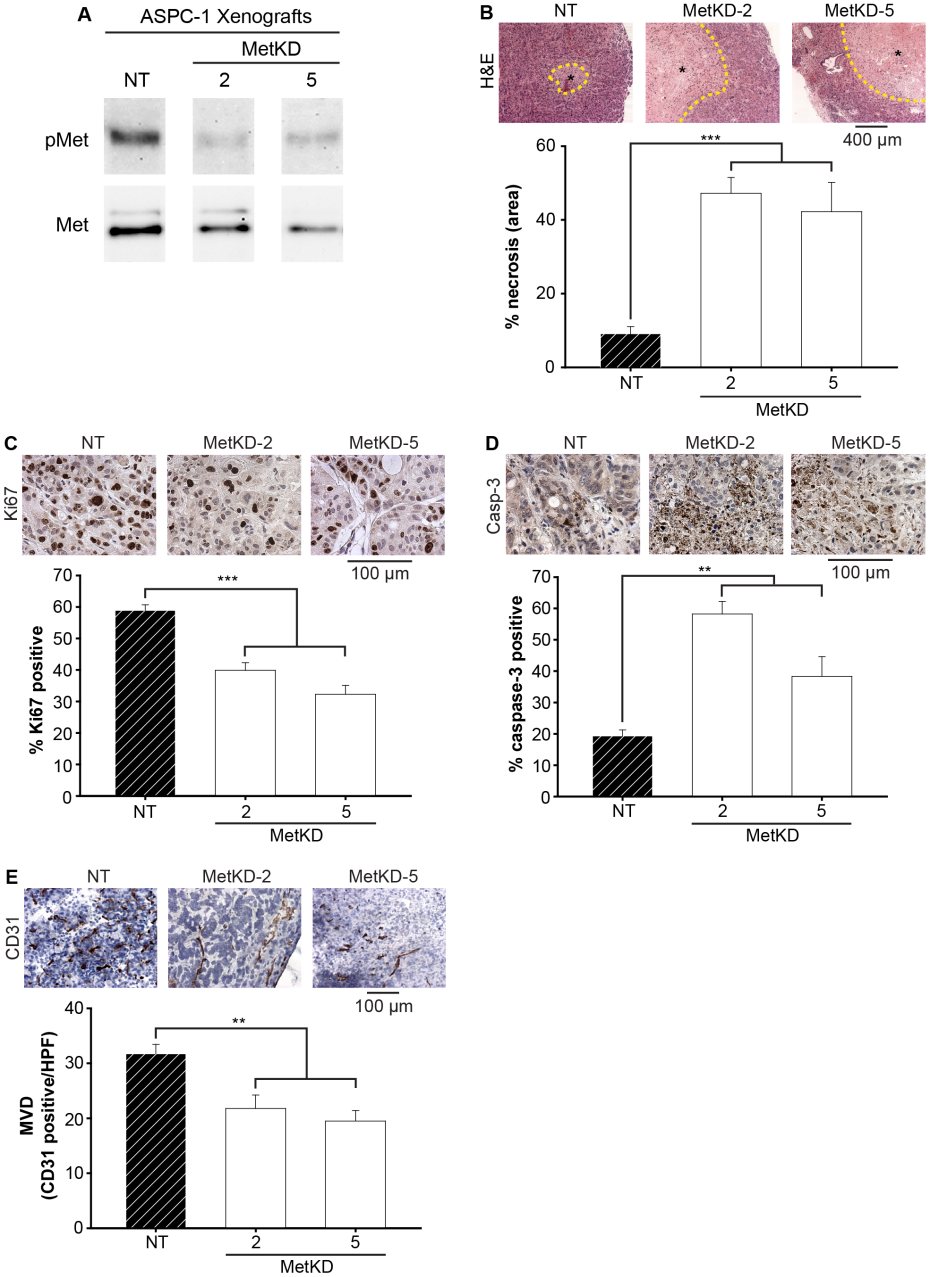
Decreased cell survival and tumor angiogenesis in ASPC-1 MetKD tumors. Immunoprecipitation and Western analysis detected strong phosphorylated Met (pMet) in NT tumors relative to low pMet levels in MetKD tumors (A). Serial sections of paraffin-embedded tumors were either H&E stained (B) or processed for Ki67 (C), active caspase-3 (D) and frozen sections for CD31 (E) staining. Morphometric analysis indicates a significant increase in necrosis and cleaved caspase-3 staining with a corresponding decrease in Ki67 and CD31 staining per high powered field (HPF) in MetKD tumors relative to NT tumors (**p<0.01, ***p<0.001; ANOVA). Representative field images are shown.

Orthotopic ASPC-1 tumors were removed and processed for further analysis. Immunoprecipitation and Western analysis of human Met in the orthotopic tumors confirmed the activation of human Met by murine HGF *in vivo* (Figure 4A), consistent with our earlier studies showing activation of human Met by recombinant mouse HGF (Refer Figure 3A & 3B). As expected lower levels of phospho-Met was detected in the Met KD tumors relative to NT xenografts (Figure 4A). Interestingly, tumors produced by ASPC-1 MetKD cells routinely contained extensive regions of central necrosis (Figure 4B) compared to the larger tumors resulting from ASPC-1 NT cells. Quantification detected a 5-fold increase in necrotic areas within the smaller tumors derived from MetKD-2 and MetKD-5 cells compared to NT control tumors (Figure 4B). The increased tumor necrosis observed in MetKD ASPC-1 xenografts could be due to rapid tumor growth that exceeds the tumor vasculature, increased tumor cell apoptosis or decreased tumor angiogenesis. To distinguish between these possibilities, we stained ASPC-1 MetKD and NT tumor sections for Ki67 or cleaved caspase-3 to assess the level of cell proliferation and apoptosis respectively. Morphometric analysis of Ki67 staining in tumors sections resulting from MetKD cells revealed fewer Ki67 positive cells per field compared to NT tumors (Figure 4C). Examining tumor sections for cleaved caspase-3 staining revealed higher numbers of cleaved caspase-3 positive cells in MetKD tumors relative to NT tumors (Figure 4D), indicating that loss of Met signaling could result in increased susceptibility to apoptosis. The increased apoptosis and necrosis observed in MetKD tumors could be attributed to decreased tumor angiogenesis. To test this, we determined the mean vessel density (MVD) in NT and MetKD-tumors by immunohistochemical staining using antibodies against CD31, a marker for endothelial cells [28]. Consistent with our data showing increased necrosis in MetKD-2 and MetKD-5 tumors, MVD was significantly reduced in the periphery of MetKD tumors compared to NT-derived pancreatic xenografts (Figure 4E). No signal was detected within the central mass of the xenograft tissues, consistent with reports on human and mouse models of pancreatic cancer [34]. Thus loss of Met signaling in ASPC-1 cells reduces tumor burden in a mouse orthotopic model, likely as a result of decreased cell survival and tumor angiogenesis.

### Met Signaling Promotes the Secretion of Angiogenic Factors

**Figure 5:**
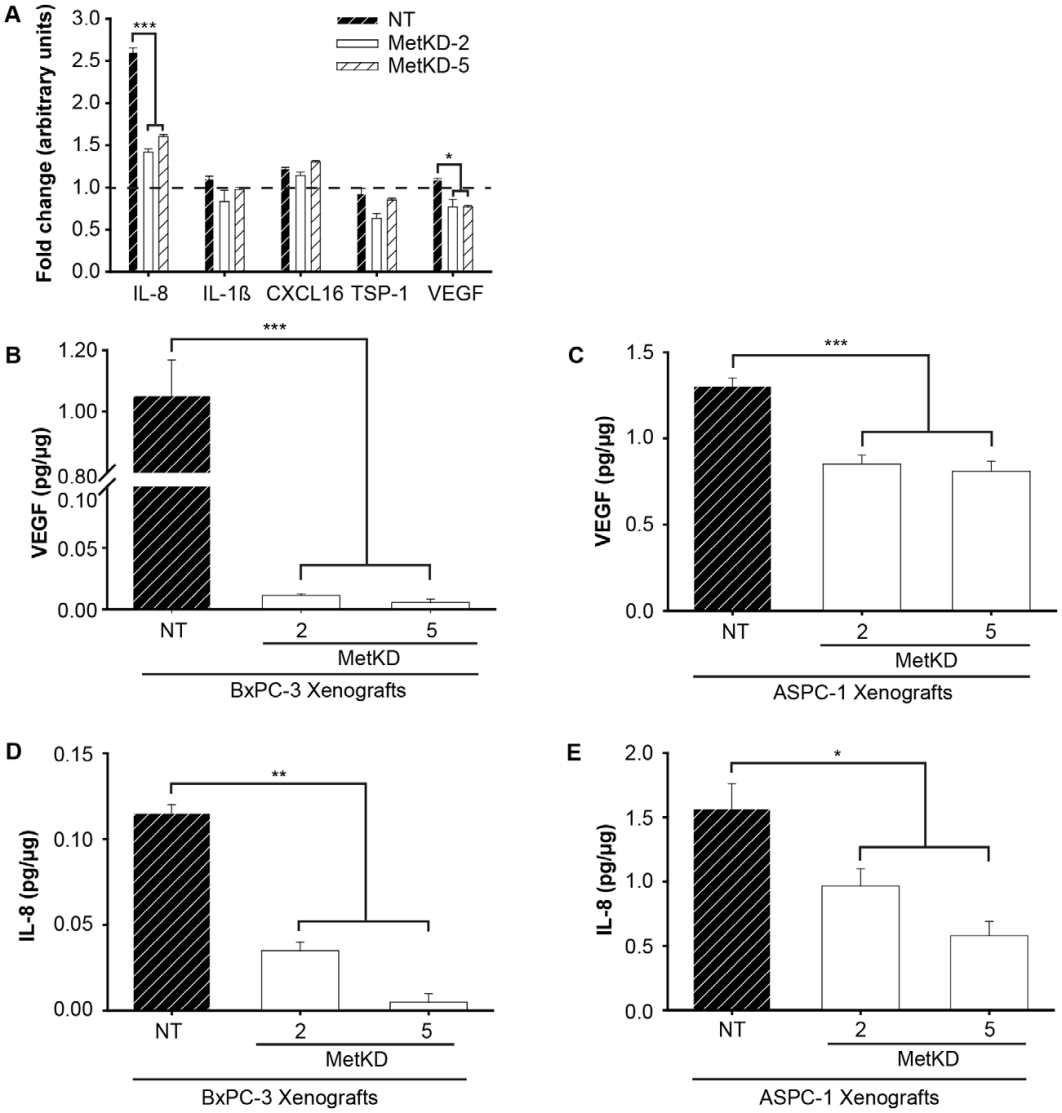
Met knockdown alters secretion of angiogenic factors. Conditioned media collected from ASPC-1 NT or MetKD cells treated with or without HGF (48 hr) was used to probe angiogenesis antibody arrays in duplicate. Data are presented as the fold difference of the indicated factor in response to HGF +/− SEM (*p<0.05, ***p<0.001; ANOVA) (A). VEGF ELISA shows decreased VEGF levels in BxPC-3 (B) and ASPC-1 (C) MetKD xenografts relative to NT control tumors (***p<0.001; ANOVA). IL-8 ELISA detected reduced IL-8 levels in BxPC-3 (D) and ASPC-1 (E) derived MetKD pancreatic xenografts compared to NT xenografts (*p<0.05, **p<0.01; ANOVA).

Met signaling has been shown to promote the remodeling of the tumor vasculature in breast and uterine tumors through the upregulation of VEGF and down regulation of thrombospondin-1 (TSP-1) [35]. VEGF is a potent agonist of angiogenesis that activates both endothelial cell proliferation and migration. In opposition, TSP-1 suppresses angiogenesis by inhibiting endothelial cell proliferation and inducing endothelial cell apoptosis. The reduced tumor vasculature detected in Met-deficient pancreatic xenografts could be the result of altered secretion of tumor-derived angiogenic factors. To test this, we probed angiogenic antibody arrays using conditioned media from ASPC-1 MetKD and NT cells (Figure 5A). No significant difference in the secretion of TSP-1 was detected in response to Met knockdown. Conversely, we detected an 18–23% reduction in the secretion of VEGF in conditioned media from MetKD cells compared to NT cells. More notably, Met knockdown resulted in a 39–42% reduction in secreted IL-8, a pro-angiogenic chemokine. As expected, HGF immunoreactivity was only observed in the HGF-treated arrays (Supporting Information S3). ELISA confirmed that Met knockdown in ASPC-1 cells reduced the secretion of IL-8 and VEGF *in vitro* (data not shown). To determine whether the reduced vasculature observed in Met-deficient tumors is due in part to decreased IL-8 and/or VEGF levels *in vivo*, we performed ELISA on proteins lysates prepared from ASPC-1- and BxPC-3-derived NT and MetKD pancreatic xenografts. Reduced VEGF levels were detected in xenografts derived from ASPC-1 MetKD cells, and to a greater extent in BxPC-3 MetKD cells when compared to control NT tumors (Figs 5C & 5B). In the case of IL-8, the level of IL-8 detected in BxPC-3 MetKD xenografts was reduced 3–10 fold relative to NT control xenografts (Figure 5D). Similarly, we detected a 2–3 fold decrease in the amount of IL-8 detected in ASPC-1-derived MetKD xenografts relative to control NT tumors (0.97+/−0.14 pg/µg and 0.58+/−0.11 pg/µg versus 1.56+/−0.20 pg/µg respectively) (Figure 5E). Thus Met knockdown in BxPC-3 and ASPC-1 cells results in decreased VEGF and IL-8 secretion *in vivo*.

**Figure 6:**
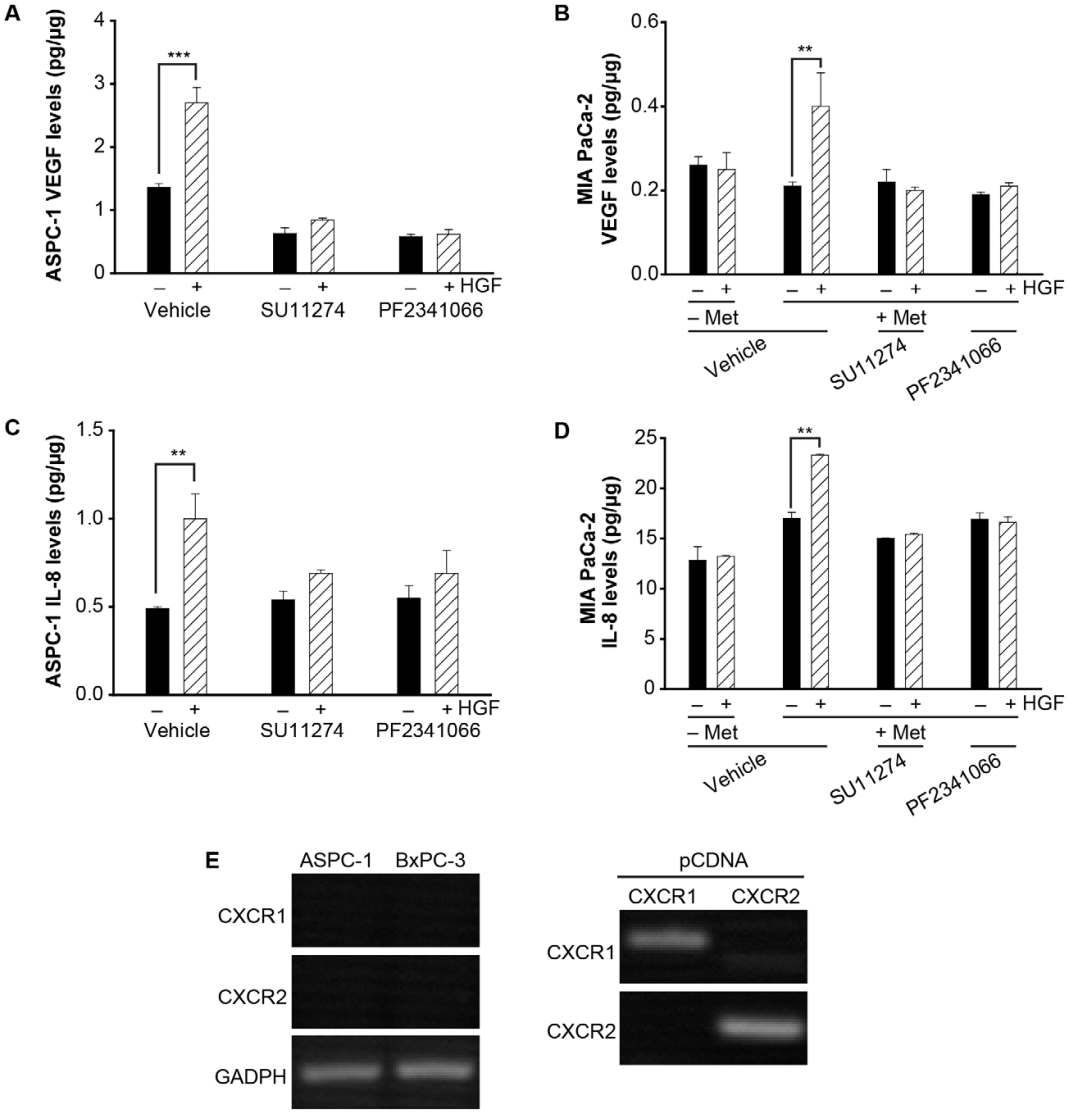
Met kinase activity regulates VEGF and IL-8 secretion. Treatment of ASPC-1 (A & C) or MIA PaCa-2 without (-Met) or stably expressing exogenous Met (+Met) (B & D) with SU11274 or PF2341066 blocked HGF-induced VEGF (A & B) and IL-8 (C & D) secretion (**p<0.01, ***p<0.001; ANOVA, n = 4). RT-PCR confirmed that ASPC-1 and BxPC-3 cells do not express the IL-8 receptors CXCR1 or CXCR2. GADPH was used as a loading control. The specificity of primer sets for human CXCR1 versus CXCR2 primers was confirmed using plasmids encoding human CXCR1 or CXCR2 respectively.

Using small tyrosine kinase inhibitors for Met, we next examined whether IL-8 secretion is dissociable from Met activation by HGF. For these studies, we used the tyrosine kinase inhibitors SU11274 and PF2341066, which preferentially target the ATP binding of Met to block HGF-induced receptor activation [36], [37]. ELISA confirmed that HGF-induced VEGF and IL-8 secretion was inhibited in ASPC-1 cells treated with 2.5 µM SU11274 or 1 µM PF2341066 (Figure 6A & 6C), conditions we have previously reported block HGF-induced Met phosphorylation in these cells [23]. To ensure that the effects are not cell type specific, we examined HGF-induced VEGF and IL-8 secretion in MIA PaCa-2 cells which lack endogenous Met, and a polyclonal population of MIA PaCa-2 cells stably over expressing Met [23]. Consistent with our studies using ASPC-1 cells, SU11274 or PF2341066 treatment blocked HGF-induced VEGF and IL-8 secretion from MIA PaCa-2 cells expressing Met (Figs 6C & 6D). As expected no HGF induced secretion of VEGF or IL-8 was detected in parental MIA PaCa-2 cells lacking Met. In addition to functioning as a paracrine inducer of endothelial migration, IL-8 has been shown to function as an autocrine survival factor for various tumor cells. IL-8 binds with high affinity to two different receptors, CXCR1 and CXCR2. To distinguish between a paracrine or autocrine role for IL-8, we used RT-PCR to examine CXCR1 and CXCR2 expression in ASPC-1 and BxPC-3 cells. As shown in Figure 6E, expression of CXCR1 and CXCR2 is not detected in these cell lines. RT-PCR performed on pcDNA plasmids encoding CXCR1 or CXCR2 as templates confirmed the specificity of the primer sets.

## Discussion

PDAC patients continue to be diagnosed with late stage disease, with adjuvant therapy being largely ineffective. Thus an urgent need exists to identify and regulate the factors responsible for PDAC as effective avenues for therapeutic intervention. Although several studies imply a role for Met signaling in PDAC [13], [18], [19], [38], [39], many important questions remain about Met’s specific mechanisms of action. In this study we used a RNA interference approach to demonstrate an important role for Met signaling for PDAC *in vivo*. Notably, both Met shRNAs were equally effective in reducing HGF-induced anchorage independent growth, migration, and invasion of BxPC-3 and ASPC-1 cells, suggesting that the effects of Met depletion are likely specific and not due to off-target effects. In our studies we detected minimal effect of Met knockdown on cell proliferation *in vitro*, although the overall tumorigenic potential of Met *in vitro* and *in vivo* was significantly reduced in BxPC-3 and ASPC-1 MetKD cells. In mice injected with ASPC-1 MetKD cells, we detected a significant reduction in local and distal tumor dissemination relative to mice injected with NT cells, a finding that may reflect the smaller size of the primary tumors routinely produced by MetKD cells. Regardless, our data identify Met as a valid therapeutic target for PDAC intervention.

As a direct result of the hypoxic tumor parenchyma of the tumor mass and the expansion of the desmoplastic stroma, pancreatic tumors are generally considered hypo-vascularized relative to normal pancreatic tissue [40]. However, sufficient tumor vascularization is required for disease progression, a process that is actively remodeled during tumor growth. Ultrasound analysis of human PDAC specimens as well as tumors formed in Kras+Tgfbr2^KO^ (an engineered mouse model for PDAC) confirm that the peripheral and invasive portions of pancreatic tumors make direct contact with blood vessels and are densely vascularized [34], [41]. An important finding of our study is that Met knockdown was accompanied by a reduction in the MVD in the tumor periphery correlating with increased tumor necrosis. Consistent with this, Tomioka and colleagues [42], [43] reported that treatment with the recombinant HGF antagonist NK4 inhibited orthotopic tumor growth and dissemination *in vivo* and decreased MVD in pancreatic tumors. However, the downstream pathways responsible for Met-mediated changes in PDAC vascularization were not elucidated in these studies. VEGF was an appealing candidate given its established role in tumor angiogenesis. Moreover, VEGF is over expressed in >90% of PDAC cases, correlating with poor patient prognosis [44]. Indeed in our studies, loss of Met signaling is associated with a 20%–60% decrease in VEGF production in Met-deficient BxPC-3 and ASPC-1 cell lines as well as MetKD xenografts relative to NT controls. Unfortunately, a phase III clinical trial using bevacizumab, a humanized antibody against VEGF approved for the treatment of colon cancer, reported minimal overall survival benefit for PDAC patients with advanced disease [45] underscoring the need to identify non-VEGF pathways important for PDAC angiogenesis.

A novel finding from our study is that Met signaling regulates secretion of the proangiogenic chemokine IL-8. HGF-induced IL-8 secretion from ASPC-1 NT cells was abrogated by Met knockdown and pharamcological inhibition *in vitro* using the tyrosine kinase inhibitors SU11274 and PF2341066. Notably, we show that Met knockdown in BxPC-3 and ASPC-1-derived xenografts reduces tumor burden *in vivo* and IL-8 secretion relative to NT control xenografts. Thus, the oncogenic effects of increased Met signaling in PDAC may be attributed in part to changes in IL-8 secretion. This view is supported by the fact that over expression of IL-8 in human pancreatic tumor cells favors tumor growth *in vivo*, whereas IL-8 antisense expression reduces tumor burden [46]. Similarly, tumor-derived IL-8 has been shown to promote angiogenesis by directly increasing endothelial cell proliferation and migration [47]. Although homologs for rat and mouse IL-8 have not been identified, IL-8 activity is not species specific. Indeed, antibodies to human IL-8 inhibit inflammation and neutrophil infiltration in rats confirming the presence of a murine IL-8 receptor. Genetically engineered mice lacking the murine IL-8 receptor homolog (mCXCR2^−/−^) show reduced neutrophil migration to sites of inflammation, as well as decreased endothelial progenitor mobilization and neo-vascularization of human melanoma xenografts relative to wild type littermates [48]. Notably, blockage of the mCXCR2 axis with the inhibitor repertaxin/SB225002 delayed PDAC tumor development in the Kras+Tgfb2^KO^ PDAC mouse model. Specifically, repertaxin/SB225002 improved overall survival of mice in the treatment group, correlating with reduced tumor angiogenesis and increased cell apoptosis [41]. Finally, a retrospective study linked increased IL-8 levels in PDAC surgical specimens with advanced T-category status and poor overall patient survival [49]. How could Met-induced changes in IL-8 secretion augment PDAC? Since ASPC-1 and BxPC-3 cells do not express CXCR1 and CXCR2, our data favors a paracrine role for IL-8 in PDAC possibly through the stimulation of endothelial cell proliferation and/or mobilization to remodel tumor vasculature. Further experimentation will be required to determine whether Met regulates IL-8 secretion directly or indirectly by crosstalk with other oncogenic signaling pathways implicated in PDAC.

In summary, PDAC is a complex disease in terms of underlying mechanisms. Our data are the first to link Met signaling with IL-8 secretion in PDAC and suggest a novel mechanism by which Met signaling could promote PDAC through remodeling of the tumor vasculature to advantage tumor growth. These findings lay the foundation for future studies directed at determining the effectiveness of combining Met and IL-8-targeted therapies for disease management in the clinical setting.

## Supporting Information

Supporting Information S1 Representative wound healing images of untreated (0 hr) and HGF-treated (9 hr) showing reduced gap closure of BxPC-3 (A) and ASPC-1 (B) MetKD cells relative to their parental and NT controls.
(TIF)

Supporting Information S2 Representative H&E stained sections of BxPC-3 NT and MetKD xenografts.
(TIF)

Supporting Information S3 Representative images of angiogenic arrays probed with conditioned media isolated from HGF-treated ASPC-1 NT versus MetKD cells.
(TIF)

Movie S1 Migration of ASPC-1 NT on collagen I-coated glass bottom culture dishes. Corresponds to Figure 2D. Time of observation was (0–120 min), with frames collected every 2 min. Display rate is 5 frames/sec.
(MOV)

Movie S2 Migration of MetKD-2 on collagen I-coated glass bottom culture dishes. Corresponds to Figure 2D. Time of observation was (0–120 min), with frames collected every 2 min. Display rate is 5 frames/sec.
(MOV)

Movie S3 Migration of MetKD-5 on collagen I-coated glass bottom culture dishes. Corresponds to Figure 2D. Time of observation was (0–120 min), with frames collected every 2 min. Display rate is 5 frames/sec.
(MOV)
