# Supplementary figures and images for: Met Receptor Tyrosine Kinase Signaling Induces Secretion of the Angiogenic Chemokine Interleukin-8/CXCL8 in Pancreatic Cancer
Source: PLoS One. 2012 Jul 17;7(7):e40420. doi: 10.1371/journal.pone.0040420 (PMC3398924; doi:10.1371/journal.pone.0040420)

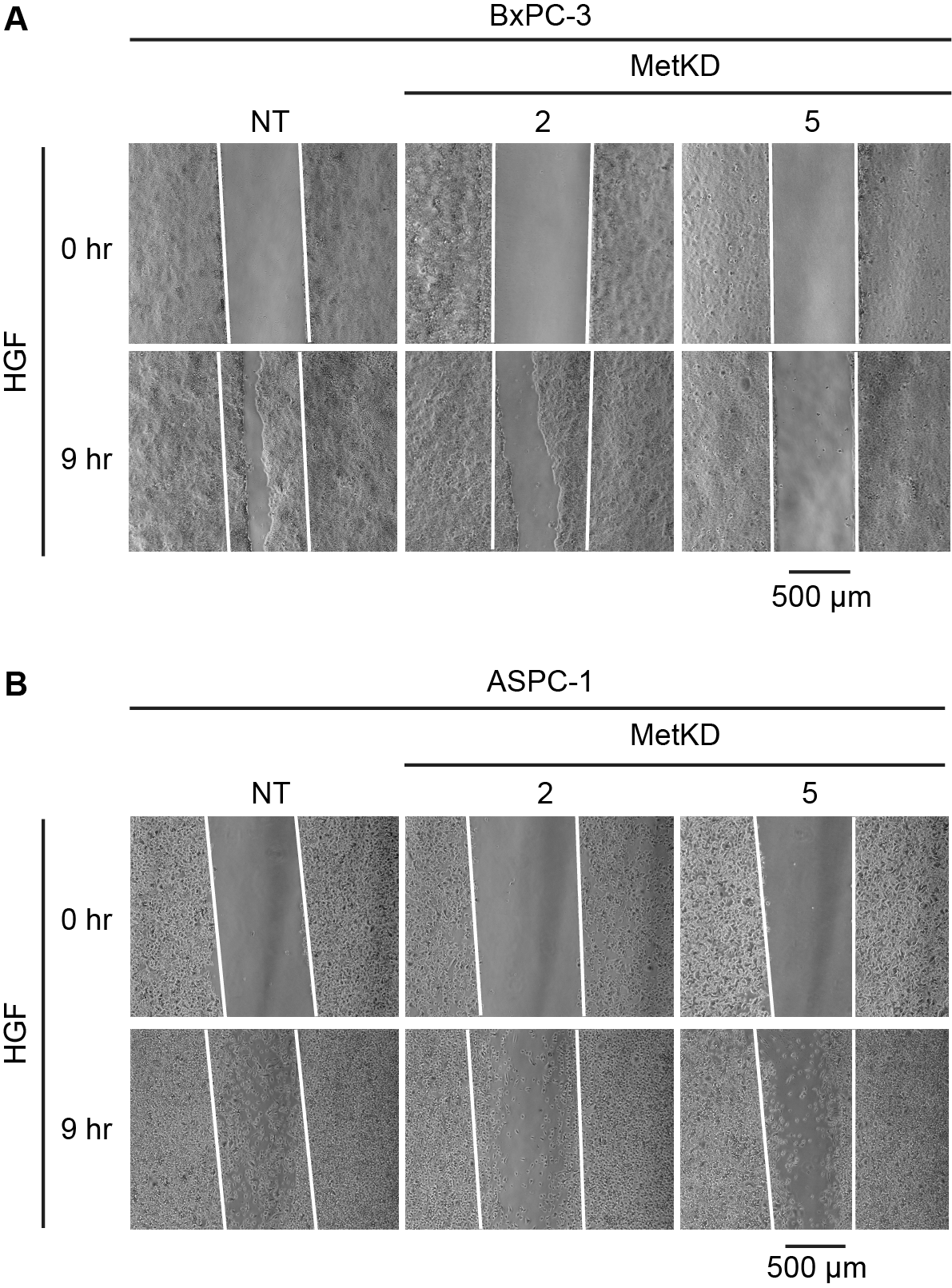

Supplement: Supporting Information S1 — Representative wound healing images of untreated (0 hr) and HGF-treated (9 hr) showing reduced gap closure of BxPC-3 (A) and ASPC-1 (B) MetKD cells relative to their parental and NT controls. (TIF) [file pone.0040420.s001.tif]

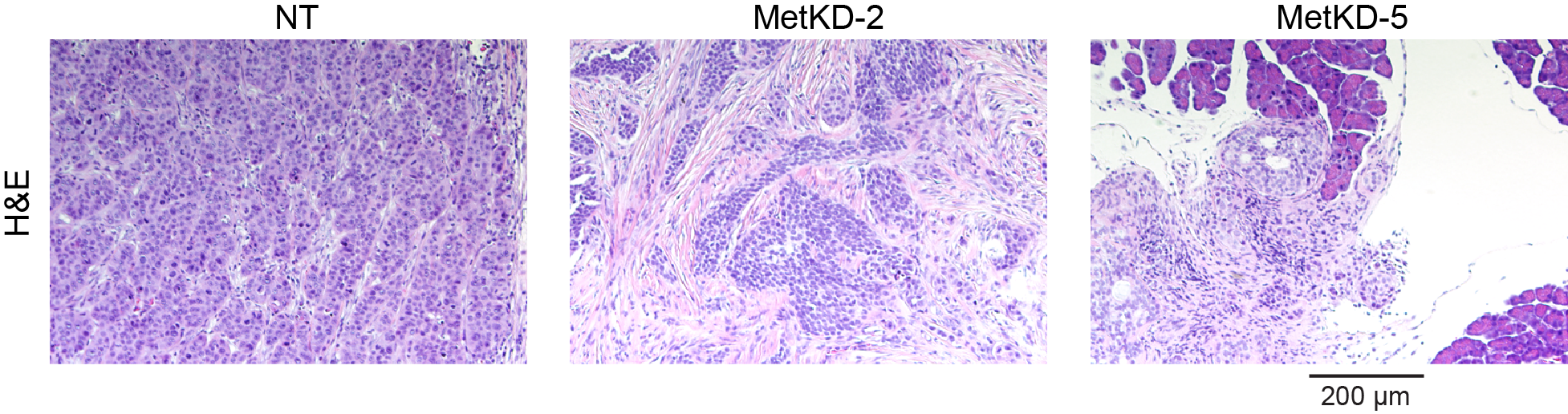

Supplement: Supporting Information S2 — Representative H&E stained sections of BxPC-3 NT and MetKD xenografts. (TIF) [file pone.0040420.s002.tif]

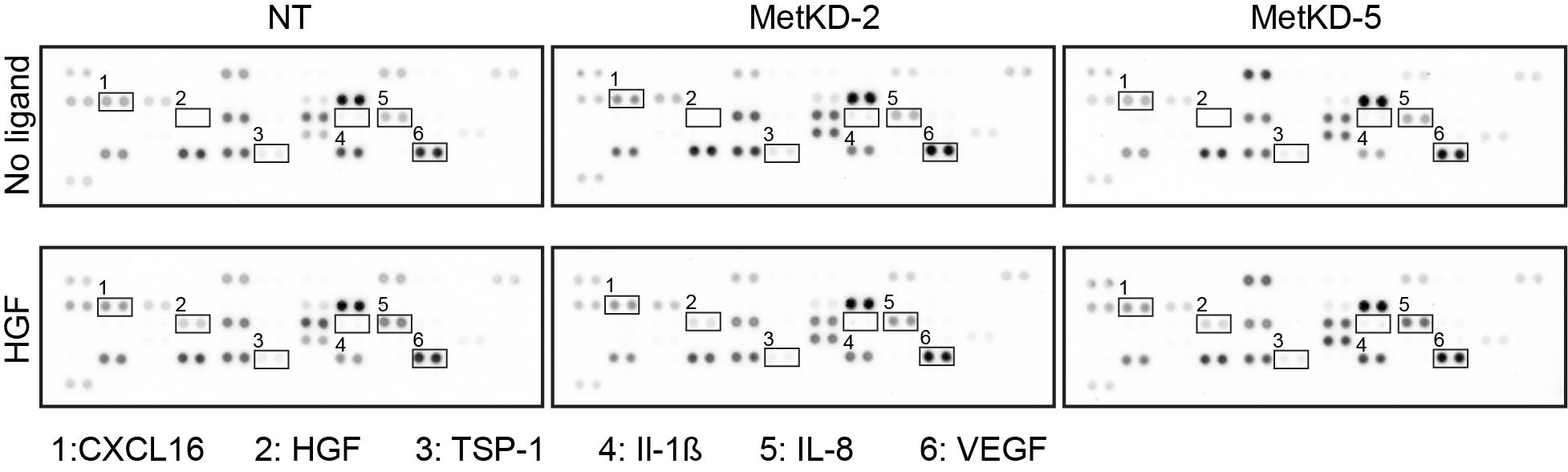

Supplement: Supporting Information S3 — Representative images of angiogenic arrays probed with conditioned media isolated from HGF-treated ASPC-1 NT versus MetKD cells. (TIF) [file pone.0040420.s003.tif]
